# Stimulation of microneedles alleviates pathology of Parkinson’s disease in mice by regulating the CD4+/CD8+ cells from the periphery to the brain

**DOI:** 10.3389/fimmu.2024.1454102

**Published:** 2024-11-19

**Authors:** Jin Hee Kim, Yujin Choi, Jin Se Kim, Hanbyeol Lee, In Gyoung Ju, Na Young Yoo, Sookie La, Do Hyeon Jeong, Changsu Na, Hi-Joon Park, Myung Sook Oh

**Affiliations:** ^1^ Department of Biomedical and Pharmaceutical Sciences, Kyung Hee University, Seoul, Republic of Korea; ^2^ Department of Oriental Pharmaceutical Science and Kyung Hee East-West Pharmaceutical Research Institute, College of Pharmacy, Kyung Hee University, Seoul, Republic of Korea; ^3^ Raphas Co. Ltd., Seoul, Republic of Korea; ^4^ Department of Acupoint and Meridian, Korean Medical College, Dongshin University, Naju, Republic of Korea; ^5^ Department of Science in Korean Medicine, College of Korean Medicine, Kyung Hee University, Seoul, Republic of Korea; ^6^ Acupuncture and Meridian Science Research Center (AMSRC), Kyung Hee University, Seoul, Republic of Korea

**Keywords:** Parkinson’s disease, microneedle, acupuncture point, peripheral immune, neuroinflammation

## Abstract

**Introduction:**

Immune dysfunction is a major cause of neuroinflammation and accelerates the progression of Parkinson’s disease (PD). Numerous studies have shown that stimulation of specific acupuncture points (acupoints) can ameliorate PD symptoms. The purpose of this study was to investigate whether attaching microneedles to acupoints would improve PD pathology by recovering immune dysfunction.

**Methods:**

The PD mouse model was induced by intrastriatal injection of 6-hydroxydopamine (6-OHDA), and microneedle patches (MPs) or sham patches (SPs) were attached to GB20 and GB34, representative acupoints for treating PD for 14 days.

**Results:**

First, the behavioral experiment showed that motor disorders induced by 6-OHDA were significantly improved by MP. Simultaneously, 6-OHDA-induced dopaminergic neuronal death and brain neuroinflammation decreased. Conversely, SP had no effect on behavioral disorders, neuronal death, or neuroinflammation. Measurement results from flow cytometry of immune cells in the brain and blood revealed a disruption in the CD4+/CD8+ ratio in the 6-OHDA group, which was significantly restored in the MP group. The brain mRNA expression of cytokines was significantly increased in the 6-OHDA group, which was significantly decreased by MP.

**Discussion:**

Overall, our results suggest that the attachment of MPs to GB20 and GB34 is a new method to effectively improve the pathology of PD by restoring peripheral and brain immune function.

## 1 Introduction

Parkinson’s disease (PD) is the second most common neurodegenerative disease, which is characterized by the primary symptoms of behavioral disorders, such as stiffness and tremors (1, 2). A reduction in dopamine levels caused by the death of dopaminergic neurons in the striatonigral circuit is a prominent pathological feature of PD (3, 4). Several mechanisms can contribute to this process, and among the mechanisms identified to date, neuroinflammation is recognized as a common pathophysiology in PD (5, 6). Neuroinflammation is a component of the immune system that protects the brain by eliminating or suppressing pathogens. This may exert beneficial effects by promoting tissue repair or disposal of cellular debris. However, persistent neuroinflammatory responses can contribute to neuronal death (6–8).

Immune dysregulation occurs both centrally and peripherally in patients with PD (5, 9). According to previous studies, immune cell dysfunction occurs in both the brain and blood of patients with PD, and damage to CD4+ T cells and hyperactivity of CD8+ T cells among lymphocytes have consistently been reported (10, 11). In this situation, the cytokines released by aberrant immune cells directly influence neuronal death and exacerbate disease progression (12). Therefore, reducing neuroinflammation by normalizing immune system regulation is a key approach for suppressing neuronal death in the treatment of PD.

Acupuncture points (acupoints) are traditionally believed to reflect the condition of organs at specific points under the skin surface (13, 14). Acupuncture and moxibustion, which stimulate specific acupoints, have long been used as treatment methods. The acupoints known to have therapeutic effects on PD include ST36, GB34, GB20 (GV16), and GV20 (15). In previous clinical studies, stimulation of these acupoints was found to significantly improve the unified PD rating scale in patients with PD (16, 17). Among these, GB34 and GB20 have been reported to suppress neuroinflammation and enhance neurogenesis, and their anti-PD effects have been demonstrated in a previous meta-analysis (16, 18, 19).

Recently, microneedle patches (MPs) have emerged as a new method for effectively stimulating acupoints (20). MPs comprise a micron-scale array of needles with a length of 25 to 2,000 µm, and has the advantage of being non-invasive, causing less pain, being easier to use, and reducing the risk of infection (21, 22). In an unpublished study, we found that the attachment of MPs to the acupoints GB34 and GB20 improved motor disorders in 1-methyl-4-phenyl-1,2,3,6-tetrahydropyridine-induced PD mice by regulating neurotransmitters and heme oxygenase-1(HO-1)/nuclear factor erythroid-2-related factor 2(Nrf2) signaling in the brain. In one prior study, Zhang et al. investigated the effects of thymopentin-containing MPs by attaching acupoints and non-acupoints to immunosuppressed mice (20). The spleens of the group with MPs attached to the acupoints showed a better immunomodulatory effect, confirming the effect of the acupoints. Based on previous studies, we hypothesized that the attachment of MPs to GB20 and GB34 would effectively stimulate acupoints and regulate immunity to improve PD pathology.

In this context, the aim of this study was to investigate whether stimulation of acupoints GB34 and GB20 using MPs could regulate the immune system from the periphery to the brain and improve the pathology of PD in PD mice. We assessed the effects of MPs attached to acupoints on behavioral disorders and histological changes in a mouse model of PD injected with 6-hydroxydopamine (6-OHDA). We further performed flow cytometry of the brain and blood to evaluate the peripheral and central immune systems.

## 2 Materials and methods

### 2.1 Materials

6-OHDA, hydrogen peroxide, bovine serum albumin (BSA), tribromoethanol, phosphate buffered saline (PBS), paraformaldehyde (PFA) and sucrose were purchased from Sigma Aldrich (St Louis, MO, United States). Rabbit anti-ionized calcium-binding adapter molecule-1 (Iba-1) was purchased from Fujifilm Wako (Chuo-Ku, Osaka, Japan). 3,3-diaminobenzidine (DAB), rabbit anti-tyrosine hydroxylase (TH) were purchased from Merck Millipore (Burlington, MA, United States). Rabbit anti-Bcl-2–associated X protein (Bax) antibody and rabbit anti-Nrf2 antibody were purchased from Abcam (Cambridge, UK). Rabbit anti-Bcl-2 antibody, mouse horseradish peroxidase (HRP)-conjugated β-actin antibody and goat anti-Glial fibrillary acidic protein (GFAP) were purchased from Santa Cruz Biotechnology (Temecula, CA, USA). Biotinylated goat anti-rabbit antibody, avidin–biotin complex (ABC), normal goat serum, streptavidin-Alexa 594 and Alexa 488 were purchased from Vector Labs (Burlingame, CA, United States). Anti-rabbit HRP secondary antibodies and rabbit anti-HO-1 antibody were purchased from Enzo Life Science, Inc. (Farmingdale, NY, USA).

### 2.2 Animals

Seven-week-old male ICR mice were purchased from Daehan Biolink (Eumseong, Republic of Korea). Mice were accommodated at a maintained condition (temperature: 23 ± 1°C, humidity: 60 ± 10% a 12 h light/dark cycle, and water and food ad libitum). All animal studies were performed in accordance with the “Guide for the Care and Use of Laboratory Animals, 8th edition” (National Institutes of Health, 2011) and approved by the “Animal Care and Use Guidelines” of Kyung Hee University, Seoul, Republic of Korea (Approval number: KHSASP-23-536).

### 2.3 Preparation of MPs

The MN patches were provided by Raphas Co. Ltd. (Seoul, Republic of Korea) and manufactured using a previously reported method (23, 24). Biocompatible microneedles were fabricated using the droplet extension method. A pharmaceutical-grade hyaluronic acid solution was dried on top of a hydrocolloid patch to form five arrays with a 1 mm base width and 350 μm height. A viscous biocompatible polymer was dropped onto the bottom layer of the patch, followed by contact with the other substrates. The two substrates were then separated by a certain distance to stretch the contacting polymer materials. After the tensioning process, drying was conducted to set the shape in the stretched state. Finally, the central part was removed to form identical microneedles on the two substrates. As a Sham patch (SP), only a patch without microneedles was used.

### 2.4 Surgical procedure for 6-OHDA injection

The injection of 6-OHDA was performed in accordance with previous studies (25, 26). Mice were anesthetized with tribromoethanol (312.5 mg/kg, i.p.) and placed on a stereotaxic apparatus. Each mouse received a unilateral injection of 2 μL vehicle (saline with 0.1% ascorbic acid, for sham-operated mice) or 6-OHDA (8 μg/μL) into the right striatum (ST) (coordinates with respect to bregma in mm: AP 0.5, ML 2.0, DV−3.0), according to the stereotaxic atlas of mouse brain (Franklin and Paxinos, 2013). 6-OHDA was delivered by a microinjection pump at an injection rate of 0.5 μl per min, and the cannula was left in place for 4 min after the end of injection.

### 2.5 Experimental design

**Figure 1:**
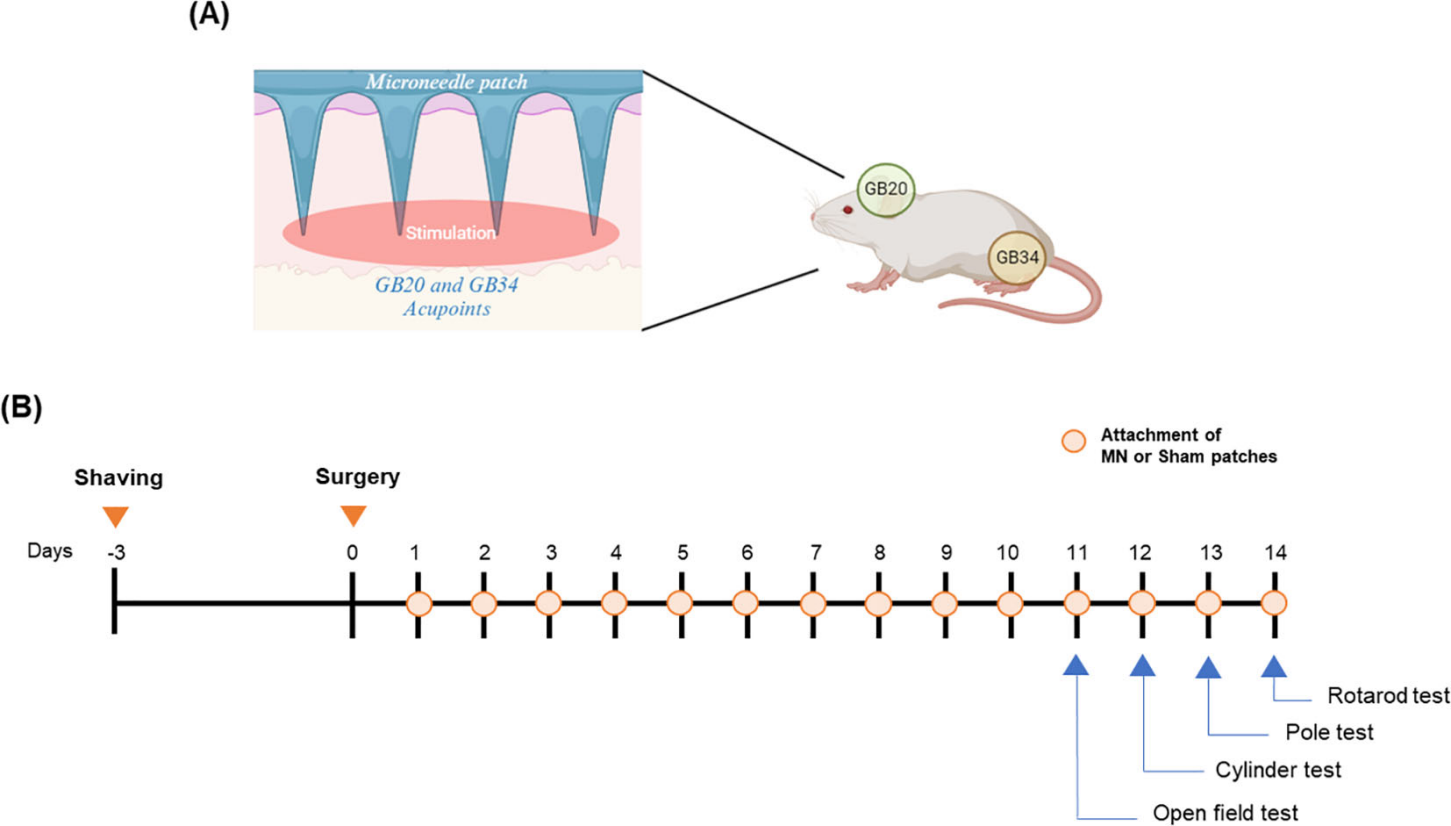
Overview of experimental procedure. Attachment points of patches on a mouse **(A)** and overall experimental schedule **(B)**.

Sixty-four male mice were used for immunohistochemistry and western blot. The mice were randomly divided into four groups as follows: 1) SHAM group (n = 17), 2) 6-OHDA group (6-OHDA-injected, n = 16), 3) SPs group (6-OHDA-injected plus attachment of SPs, n = 15), 4) MPs group (6-OHDA-injected plus attachment of MPs, n = 16). Seven to nine mice per group were sacrificed for immunohistochemistry, and the remaining mice were sacrificed for western blot. Forty male mice were used for flow cytometry and quantitative reverse transcription-polymerase chain reaction (qRT-PCR). The mice were randomly divided into four groups as follows: 1) SHAM group (n = 9), 2) 6-OHDA group (6-OHDA-injected, n = 9), 3) SPs group (6-OHDA-injected plus attachment of SPs, n = 9), 4) MPs group (6-OHDA-injected plus attachment of MPs, n = 9). Five mice per group were sacrificed for flow cytometry, and the remaining mice were sacrificed for mRNA extraction. Before 6-OHDA surgery, the acupoint area was shaved to attach the patches. In the SPs or MPs group, patches were attached to the GB20 and GB34 areas for a total of 14 days, starting one day after 6-OHDA injection (**Figure 1**). The patch was attached for 2 h, and behavioral experiments or sacrifice were conducted 1 hour after the patch was removed.

### 2.6 Behavioral tests

#### 2.6.1 Pole test

Pole test was performed on mice for immunohistochemistry and western blot analyses. Prior to the test, the mice underwent a single training session. During the test, each mouse was placed at the top of a pole (diameter = 8 mm, height = 55 cm, rough surface) facing upward. The times required for head down (T-turn) and landing (T-LA) were recorded. If a mouse failed to descend and fell immediately, a maximum time of 60 s was assigned. Three trials were conducted for each mouse, and the average of these trials was calculated (27).

#### 2.6.2 Rotarod test

Rotarod test was performed on mice for immunohistochemistry and western blot analyses. The rotarod device contained a rotating spindle (30 mm in diameter) and five separate compartments for the simultaneous examination of five mice. The rotarod tests were performed for 3 min at a constant speed between 10 and 12 rpm. The training was conducted by placing the mouse on a spindle whenever it fell on the ground. During the test session, the test was performed at the same rotational speed as that of the training. The time that remained on the rotating spindle until the first drop (latency time) was recorded. If no fall occurred, a maximum time of 180 s was assigned. The test was repeated three times, and the average latency time was calculated (27, 28).

#### 2.6.3 Cylinder test

Cylinder test was performed as previously study on mice for immunohistochemistry and western blot analyses (29). Brifly, each mouse was placed in a clear cylinder (8.5 cm diameter, 12 cm height) for 5 min without any habituation prior to recording. The use of the ipsilateral forelimb, contralateral forelimb, or both forelimbs during rat rearing was recorded by a blinded examiner. The results are presented in terms of the ratio of contralateral forelimb (left) wall touches relative to the number of touches by ipsilateral forelimb (right).

#### 2.6.4 Open field test

OFT was performed on mice flow cytometry and qRT-PCR. For the OFT, the mice were placed in a box (45 × 45 × 45 cm). The central area of the box measured 15 cm × 15 cm. After placing the mouse in the box, the first 5 min was not recorded, and then the position of the mouse was analyzed using an automated computer analysis system (Biobserve, Bonn, Germany) during the 10 min test (30).

### 2.7 Tissue preparation

On day 14 of SP and MP attachment, mice were anesthetized 1 h after patch removal. Mice for immunohistochemistry were transcardially perfused with 0.05 M PBS, and subsequently fixed with pre-chilled 4% PFA in 0.1 M phosphate buffer. Whole brain tissues were post-fixed with 4% PFA overnight, immersed in a solution containing 30% sucrose in 0.05 M PBS, and stored at 4°C until sectioning. The frozen brains were coronally sectioned on a cryostat at 25 μm, after which they were stored in a storage solution at 4°C. The mice for western blot and qRT-PCR were decapitated, and the ST and substantia nigra (SN) in their vehicle or 6-OHDA injected right hemisphere was isolated and stored at – 80°C until. All tissues used were from the hemisphere injected with 6-OHDA or vehicle only. Half of the ST was used as tissue for measuring dopamine levels, and the other half was used for western blot.The mice used for flow cytometry were anesthetized, and blood was collected from the heart. The right hemisphere of the brain was prepared and injected with vehicle or 6-OHDA. Flow cytometry was performed immediately on the fresh tissue.

### 2.8 Immunohistochemistry

Brain sections were rinsed in 0.05 M PBS and incubated with 1% hydrogen peroxide (H_2_O_2_) in 0.05 M PBS for 15 min. Subsequently, sections were replaced with anti-TH antibody (Cat no.: AB152, 1:1000) or anti-DAT antibody (Cat no.: MAB369, 1:1000) in 0.3% Triton X-100, 1% normal goat serum in 0.05 M PBS overnight at 4°C. They were subsequently incubated in an ABC solution with biotinylated anti-rabbit immunoglobulin G (IgG) or anti-rat IgG antibodies (Cat no.: BA-1000, BA-4000, 1:500). For immunofluorescence staining, brain sections were rinsed with 0.05 M PBS, and subsequently incubated with blocking buffer (0.05 M PBS containing 1% BSA, 3% normal goat serum, and 0.4% Triton X-100) for 1 h at room temperature (RT). The sections were incubated with the anti-Iba-1 antibody (Cat no.: 019-19741, 1:1000) or anti-GFAP antibody (Cat no.: SC-6170, 1:2000) overnight at 4°C in the blocking buffer. After rinsing with 1X PBS, cells were visualized with anti-goat Alexa Fluor 594 (Cat no.: A21468, for GFAP) or anti-rabbit Alexa Fluor 488 (Cat no.: A11008, for Iba-1) diluted in blocking buffer (1:500) for 1 h at RT. Images were captured using a microscope (K1-Fluo confocal microscope (Nanoscope Systems, Daejeon, Korea) or Olympus BX51 Fluorescence Microscope (Olympus, Tokyo, Japan). The areas or numbers of TH-, DAT-, Iba-1-, and GFAP-positive cells were analyzed using ImageJ software (National Institutes of Health, Bethesda, MD, USA). All values were calculated by averaging three tissue sections per mouse.

### 2.9 Western blot

SN tissues were lysed in RIPA buffer containing a protease/phosphatase inhibitor cocktail. Proteins were separated using sodium dodecyl sulfate-polyacrylamide gel electrophoresis and transferred onto polyvinylidene fluoride membranes. The membranes were blocked with 5% BSA for 30 min, then incubated at 4°C with primary antibody diluted in 1% BSA overnight (TH (Cat no.: AB152) 1:1000; Bcl-2 (Cat no.: SC-783) 1:500; Bax (Cat no.: AB7977) 1:500; Nrf2 (Cat no.: AB31163) 1:500; HO-1 (Cat no.: ADI-SPA-895-F) 1:500; β-actin-HRP (Cat no.: SC-47778HRP) 1:3000). After washing with Tris-buffered saline (10 mM Tris-HCl, 150 mM NaCl, pH 7.5) containing 0.1% Tween 20, the membrane was incubated with a secondary antibody (Cat no.: ADI-SAB-300, 1:3000) at room temperature for 1 h. Proteins were detected using ECL reagent, and visualization and quantification of bands were performed using Image Lab Software (Bio-Rad, CA, USA).

### 2.10 Measurement of dopamine level

Dopamine levels were measured using a dopamine kit (KA3838, Abnova, Taipei, Taiwan) in half of the ST samples. Tissues were homogenized in 1 mM EDTA and 4 mM sodium metabisulfite to measure dopamine. Subsequently, samples were centrifuged at 12,000 rpm for 20 min and the supernatant was collected for subsequent DA measurements, performed according to the manufacturer’s instructions. This assay involved three key steps: extraction, acylation, and enzymatic analysis. Initially, dopamine was extracted from the cell lysate by adding samples to the wells of an extraction plate along with ultrapure water and TE buffer, followed by incubation at room temperature for 60 min with shaking at approximately 600 rpm. After rinsing the plate to remove excess TE buffer, acylation was performed by adding the acylation buffer and reagent to the dried extraction plate and incubating for 15 min at room temperature. The plate was then washed with wash buffer and blotted prior to adding hydrochloric acid to each well, followed by a 10 min incubation at room temperature with shaking. Next, the acylated samples were transferred to new wells, and an enzyme solution was added. The plate was incubated at 37°C for 2 h on a shaker. After this incubation, the supernatant was moved to precoated dopamine microtiter strips, and dopamine antiserum was added to each well. The plate was incubated at 2 to 8°C for 20 h, then washed thoroughly with wash buffer. The enzyme conjugate was added to all wells and incubated for 30 min with shaking. Following another wash, a substrate solution was introduced to the wells and incubated for 25 min. Finally, a stop solution was added, and absorbance due to dye development was measured at 450 nm with a reference wavelength of 630 nm using a microplate reader (31).

### 2.11 Flow cytometry

Cells from the brain were isolated for flow cytometry as previously described (32). The experimental method for preparing cells from blood for flow cytometry was as follows: fresh blood exceeding 0.6 ml was immediately placed in a heparin tube and reacted on a shaker for 20 min. Subsequently, the suspension was filled with 0.05M PBS up to 1 ml and then slowly dispensed onto the pre-dispensed histopaque. After centrifuging at 400xg for 30 min, the separated peripheral blood mononuclear cell layer was carefully obtained. To this, 200 ul of 2% FBS was added and centrifuged again at 400xg for 10 min to remove the supernatant. Then, 900 μL of red blood cell lysis buffer was added to the remaining pellet, which was reacted for 6 min, and centrifuged at 400xg for 4 min. After removing the supernatant, cells were washed with FACS buffer (containing 1% BSA and 1mM EDTA in sterile 1×PBS) at 400×g for 5 min at 4°C. Cells isolated from the brain and blood were stained with rat anti-mouse CD45R/B220, CD4, CD8a, or CD3. After incubation for 1 h at 4°C, fluorescence data were collected on a Cyto FLEX (Beckman Coulter, Brea, CA, USA) using CytExpert software (Beckman Coulter).

### 2.12 mRNA extraction and qRT-PCR analysis

Total RNA from tissues was extracted following a previously described method (33). Briefly, Trizol was added to the tissue to lyse and dissolve the cell and nuclear membranes. Following this, chloroform to the mixture, which was then centrifuged to achieve phase separation. The upper aqueous phase, containing the RNA, was carefully collected. RNA precipitation was induced by the addition of isopropanol, followed by a 75% ethanol wash to purify the RNA. The purified RNA was used to synthesize cDNA with TOPscript™ RT DryMIX (Enzynomics, Republic of Korea) for qRT-PCR. RT-PCR was performed using TOPreal™ qPCR 2X PreMIX (SYBR Green; Enzynomics, Republic of Korea), and the CFX Connect Real-Time PCR System (Bio-Rad Laboratories, USA). Primers, synthesized at Cosmo Genetech (Republic of Korea), were as follows: interleukin (IL)-6: forward, 5-CCGGAGAGGAGACTTCACAG-3, reverse, 5-TTGCCATTGCACAACTCTTT-3; IL-1β: forward, 5-CCCAAGCAATACCCAAAGAA-3, reverse, 5-GCTTGTGCTCTGCTTGTGAG-3; IL-2: forward, 5-AGGAACCTGAAACTCCCCAG-3, reverse, 5-AAATCCAGAACATGCCGCAG-3; IL-4: 5-TCTCGAATGTACCAGGAGCC-3, reverse, 5-ACCTTGGAAGCCCTACAGAC-3; IL-17a: forward, 5-GCCCTCAGACTACCTCAACC-3, reverse, 5-ACACCCACCAGCATCTTCTC-3.

### 2.13 Statistical analysis

Differences among the groups were analyzed statistically by one-way analysis of variance (ANOVA) followed by Dunnett’s *post-hoc* test or student’s *t* test using GraphPad Prism 8.0.1 software (GraphPad Software Inc., USA). All values are presented as mean ± standard error of the mean (S.E.M.). The differences were considered statistically significant at *p* < 0.05 and are expressed in each figure.

## 3 Results

### 3.1 MP attachment to acupoints attenuate 6-OHDA-induced PD symptoms in mice

**Figure 2:**
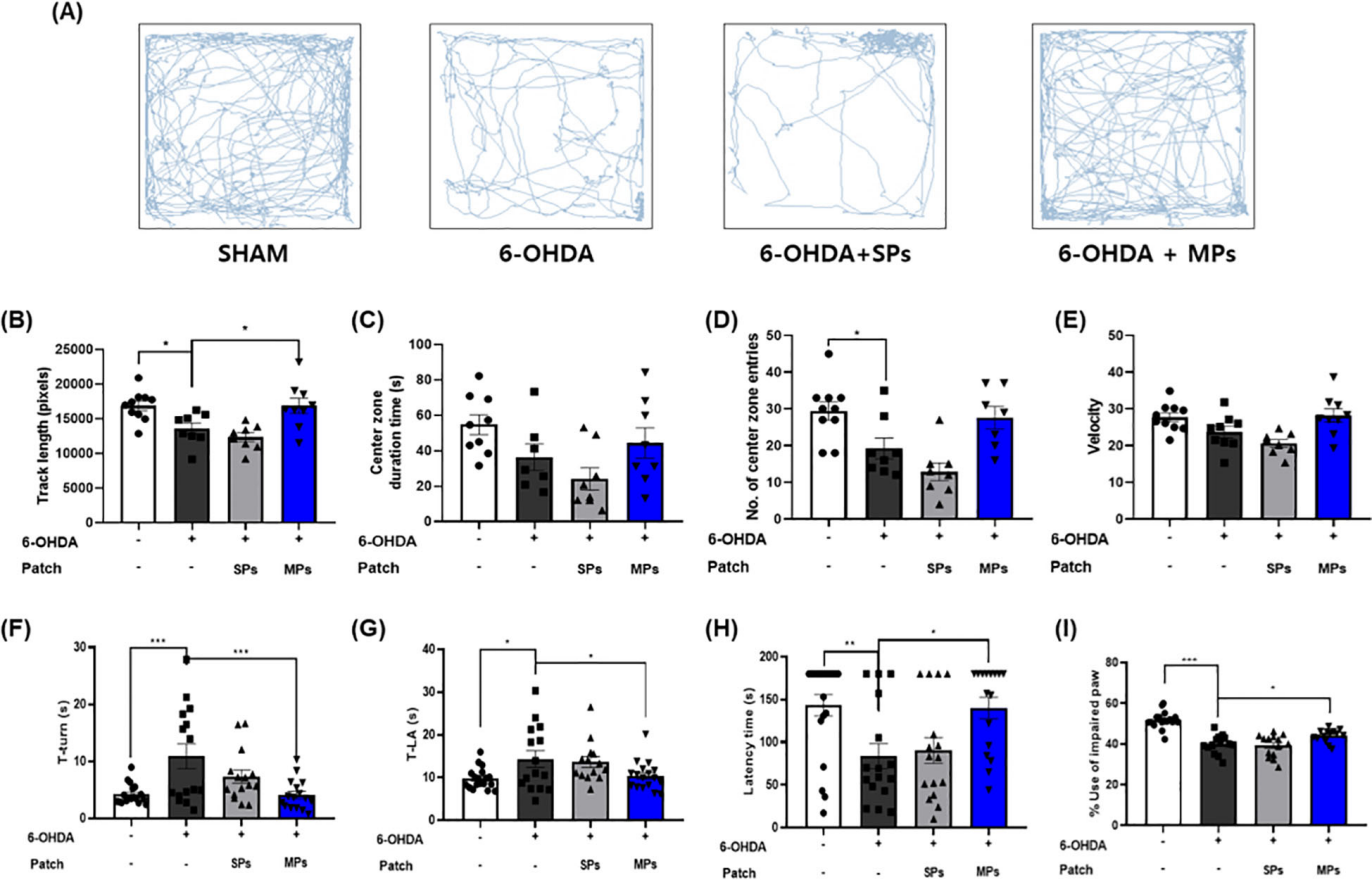
Effects of MPs attached to acupoints on PD symptoms in 6-OHDA-injected mice. Behavioral disorders were assessed using the OFT, pole test, rotarod test, and cylinder test. Representative images of the OFT are shown in **(A)**, track length **(B)**, center zone duration time **(C)**, number of center zone entries **(D)**, and velocity **(E)** were measured. T-turn and T-LA in the pole test **(F, G)**, latency time in the rotarod test **(H)**, and impaired paw usage rate in the cylinder **(I)** test were measured. Values are given as the mean ± S.E.M. Data were analyzed by One-way ANOVA followed by *post hoc* Dunnett’s multiple comparisons test. *p < 0.05, **p < 0.01 and ***p < 0.001 compared to the 6-OHDA group.

The OFT, pole test, rotarod test, and cylinder test were performed to investigate the effect of MPs attached to acupoints on PD behavioral disorders. In all behavioral experiments, motor impairment was significantly induced by 6-OHDA injection. In the OFT, track length (*p < 0.05), center zone duration (p = 0.76), and number of center zone entries (p = 0.12), and velocity (p = 0.09) increased in the MPs group compared to the 6-OHDA group (**Figures 2A–E**). In the MPs group, both T-turn (***p < 0.001) and T-LA (*p < 0.05) were significantly decreased compared to those in the 6-OHDA group in the pole test (**Figures 2F, G**). In the rotarod test, the latency time was decreased by 6-OHDA and significantly increased in the MPs group (*p < 0.05; **Figure 2H**). In the cylinder test, the use of impaired paws was significantly higher in the MPs group than in the 6-OHDA group (*p < 0.05; **Figure 2I**). However, in all behavioral experiments, the SPs group showed no significant improvement in behavioral disorders.

### 3.2 MP attachment to acupoints protects dopaminergic neurons in SN of 6-OHDA-induced PD mice

**Figure 3:**
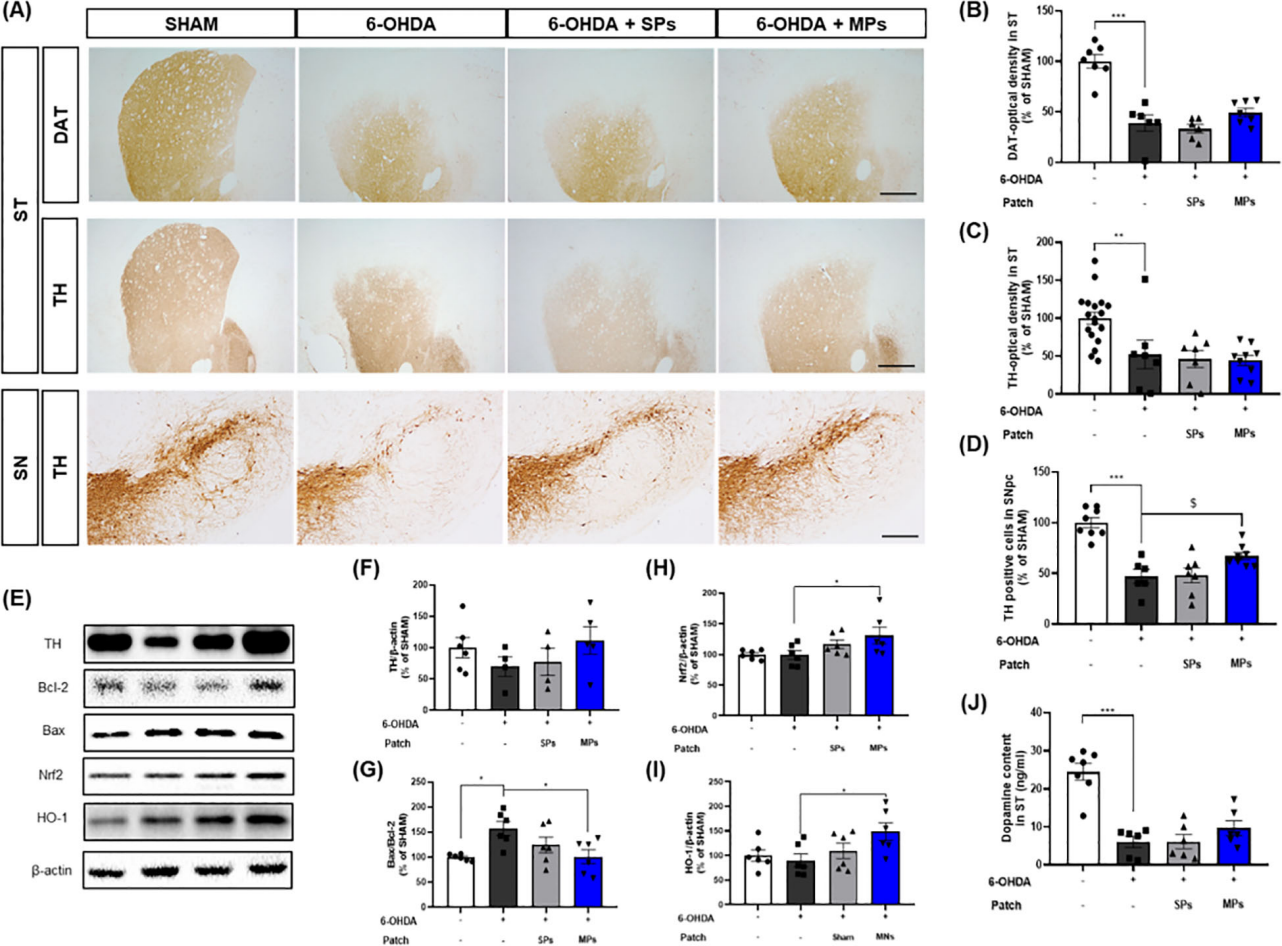
Effects of MPs attached to acupoints on dopaminergic neurons in ST and SN of 6-OHDA-injected mice. Representative images of DAT in ST (scale bar = 500 μm) and TH in ST (scale bar = 500 μm) and SN (scale bar = 200 μm) are shown in **(A)**. Graphs were represented as optical density of DAT-immunoreactivity in ST **(B)**, TH-immunoreactivity in ST **(C)** and SN **(D)**. Representative band images of TH, Bcl-2, Bax, Nrf2, and HO-1 in SN are shown in **(E)**. Graphs were represented as expression of TH **(F)**, Bcl-2/Bax ratio **(G)**, Nrf2 **(H)**, and HO-1 **(I)** in SN and dopamine content in ST **(J)**. Values are given as the mean ± S.E.M. Data were analyzed by One-way ANOVA followed by *post hoc* Dunnett’s multiple comparisons test or Student’s *t*-test. *p < 0.05, **p < 0.01 and ***p < 0.001 compared to the 6-OHDA group; $*p* < 0.05 using student’s *t*-test.

To evaluate the protective effect of acupoint-attached MPs against dopaminergic neuron loss, immunohistochemistry was performed to measure the optical density of DAT and TH in the ST and the number of TH + cells in the SN (**Figure 3A**). First, 6-OHDA injection significantly induced the loss of dopaminergic neurons in the ST (DAT, ***p < 0.001; TH, **p < 0.01) and SN (TH, ***p < 0.001). The optical densities of DAT and TH in the ST region showed no significant change owing to the attachment of SP (DAT, p = 0.86; TH, p = 0.99) or MPs (DAT, p = 0.48; TH, p = 0.99; **Figures 3B, C**). However, the number of TH+ cells measured in the SN increased in the only MPs group compared to the 6-OHDA group ($p < 0.05 in Student’s *t*-test; **Figure 3D**). These results were consistent with the protein levels in the SN (**Figure 3E**). There were no significant differences in any of the values between the SPs and 6-OHDA groups. However, the protein level of TH was reduced by 6-OHDA injection, whereas MPs enhanced it (p = 0.33; **Figure 3F**). Moreover, the ratio of Bax and Bcl-2, factors related to apoptosis, increased in the 6-OHDA group and was significantly lowered by MP attachment (*p < 0.05; **Figure 3G**). The expression levels of Nrf2 and HO-1, which are involved in cell survival through regulation of the oxidative stress response, remained unchanged following 6-OHDA injection but were significantly enhanced by the attachment of MPs to acupoints (*p < 0.05; **Figures 3H, I**). Finally, measurement of the dopamine contents in the ST revealed decreases induced by 6-OHDA, which tended to increase in the MPs group (p = 0.44; **Figure 3J**).

### 3.3 MP attachment to acupoints reduced neuroinflammation in the brain of 6-OHDA-induced PD mice

**Figure 4:**
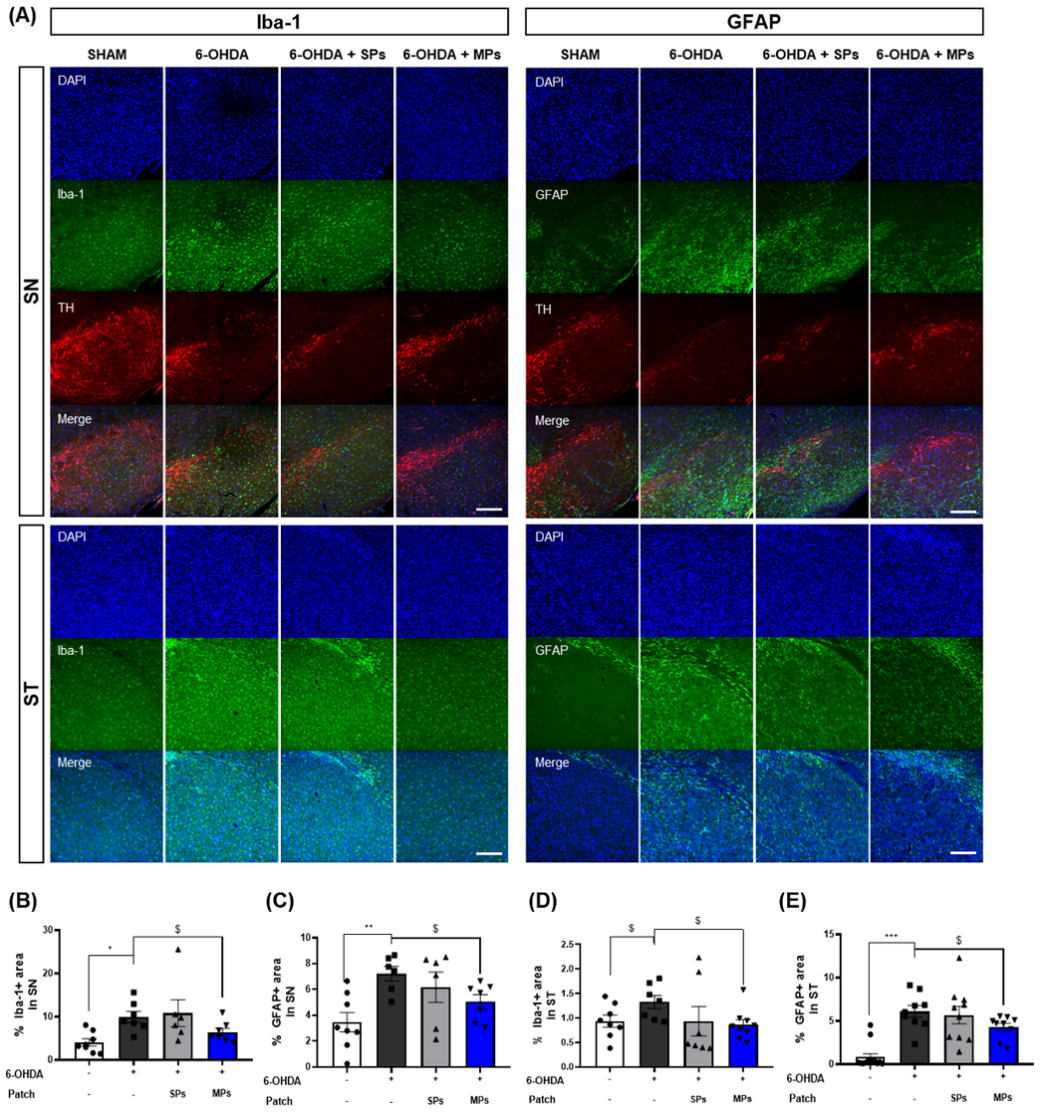
Effects of MPs attached to acupoints on expression of Iba-1 and GFAP in ST and SN of 6-OHDA-injected mice. Representative images of Iba-1 and GFAP in SN and ST (scale bar = 200 μm) are shown in **(A)**. Graphs were represented as % Iba-1+ area in SN **(B)** and ST **(D)**. Graphs were represented as % GFAP+ area in SN **(C)** and ST **(E)**. Values are given as the mean ± S.E.M. Data were analyzed by One-way ANOVA followed by *post hoc* Dunnett’s multiple comparisons test or Student’s *t*-test. *p < 0.05, **p < 0.01 and ***p < 0.001 compared to the 6-OHDA group using One-way ANOVA; $*p* < 0.05 using student’s *t*-test.

The inhibitory effect of the attachment of MPs to acupoints on neuroinflammation was evaluated through immunofluorescence for Iba-1 and GFAP, which are markers of microglia and astrocytes, in the SN and ST. First, Iba-1 staining revealed that the area of Iba-1+ cells was elevated in the 6-OHDA group (SN, *p <0.05; ST, $p< 0.05 in Student’s *t*-test). The area of Iba-1+ cells was significantly decreased in the MPs group compared to the 6-OHDA group in both the SN ($p< 0.05 in Student’s *t*-test) and ST ($p< 0.05 in Student’s *t*-test). Consistent with the results for Iba-1, the area of GFAP+ cells in the SN (**p < 0.01) and ST (***p < 0.001) increased in the 6-OHDA group, and the MPs group showed a significant reduction in GFAP+ cell area compared to the 6-OHDA group (SN and ST, $p< 0.05 in Student’s *t*-test). No significant neuroinflammatory effects were observed in the SPs group (**Figure 4**).

### 3.4 MP attachment to acupoints balancing central immune system in the brain of 6-OHDA-induced PD mice

**Figure 5:**
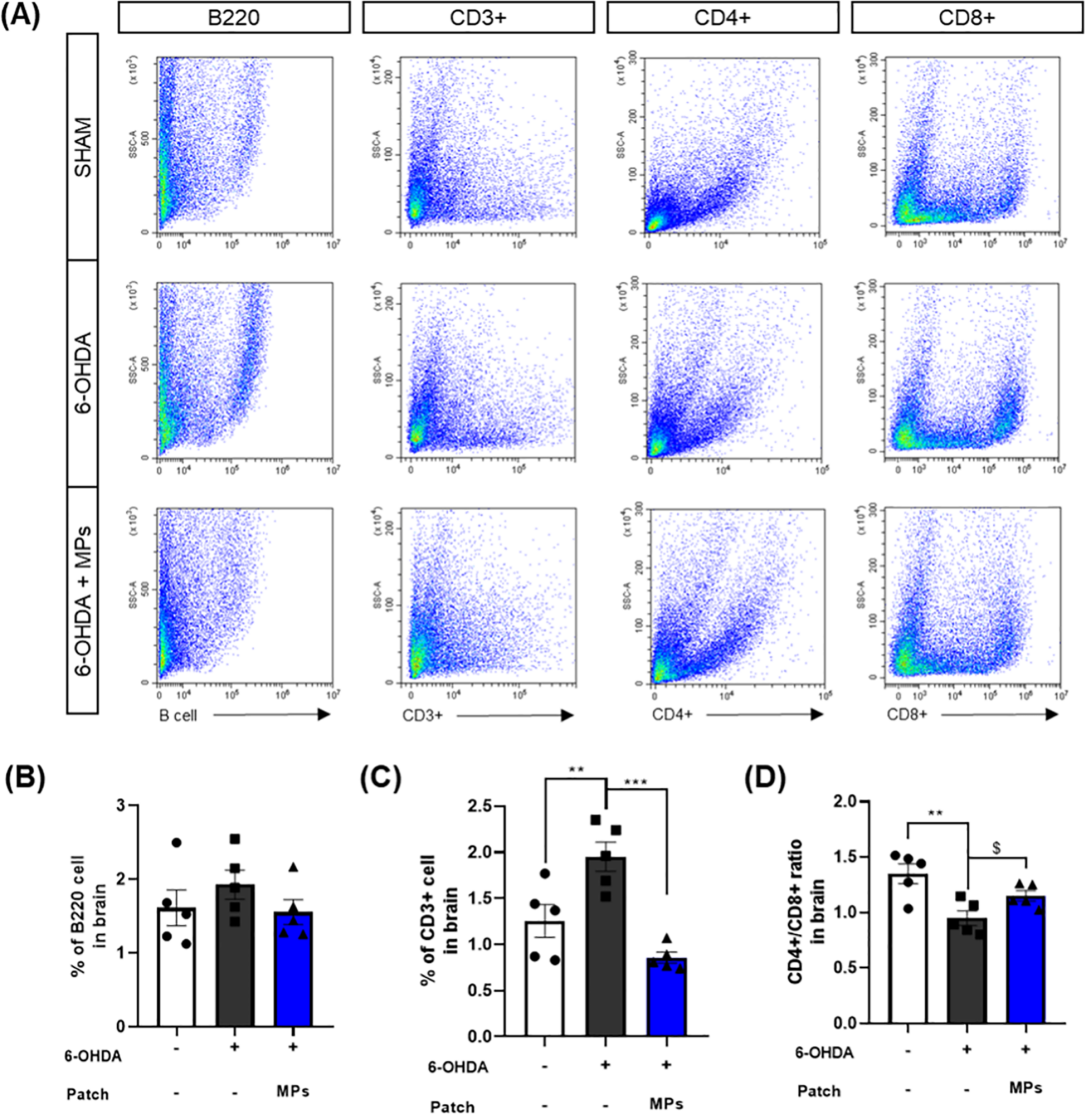
Effects of MPs attached to acupoints on % of B220, CD3+, CD4+, and CD8+ cells in the brain of 6-OHDA-injected mice. Representative flow cytometry plots of B220, CD3+, CD4+ and CD8+ cells in brain are shown in **(A)**. Graphs were represented as % of B220 **(B)**, CD3+ **(C)** and CD4+/CD8+ ratio **(D)** in brain. Values are given as the mean ± S.E.M. Data were analyzed by One-way ANOVA followed by *post hoc* Dunnett’s multiple comparisons test or Student’s *t*-test. **p < 0.01 and ***p < 0.001 compared to the 6-OHDA group using One-way ANOVA; $*p* < 0.05 using student’s *t*-test.

Immunity in PD has primarily been investigated in relation to T cells (34). Increased CD3+ T cells infiltration into the brain and a decreased CD4+/CD8+ ratio have been observed in postmortem brain tissue (10). B cells in the brain have only recently begun to be investigated in PD; however, it is believed that they may contribute to neuroinflammation because they are activated by T cells. In addition, the 6-OHDA-induced mouse model used in the present study is recognized for exhibiting an impaired blood-brain barrier (BBB) and a compromised immune system (35, 36). Therefore, to assess the effect of MPs attached to acupoints on the central immune system, we evaluated several key immune markers, including B220 and CD3+, and the CD4 +/CD8 + ratio in the hemisphere injected with 6-OHDA. First, the percentage of B220, a B cell marker, decreased in the MPs group compared to that in the 6-OHDA group (p = 0.36), although the difference was not significant. In addition, CD3+ T cells, which are the co-receptors of T cells, were investigated. The percentage of CD3+ T cells was elevated in the 6-OHDA group (**p < 0.01) and significantly lower in the MPs group than in the 6-OHDA group (***p < 0.001). Finally, the ratios of helper and killer T cells to T cells were measured. The injection of 6-OHDA significantly reduced the CD4+/CD8+ ratio (**p < 0.01), which was restored by the attachment of MPs to acupoints ($p< 0.05 in Student’s *t*-test; **Figure 5**). Therefore, we surmised that the attachment of MPs to acupoints restored the central immune system disturbed by 6-OHDA injection.

### 3.5 MP attachment to acupoints modulating mRNA expressions of cytokines in the ST and SN of 6-OHDA-induced PD mice

**Figure 6:**
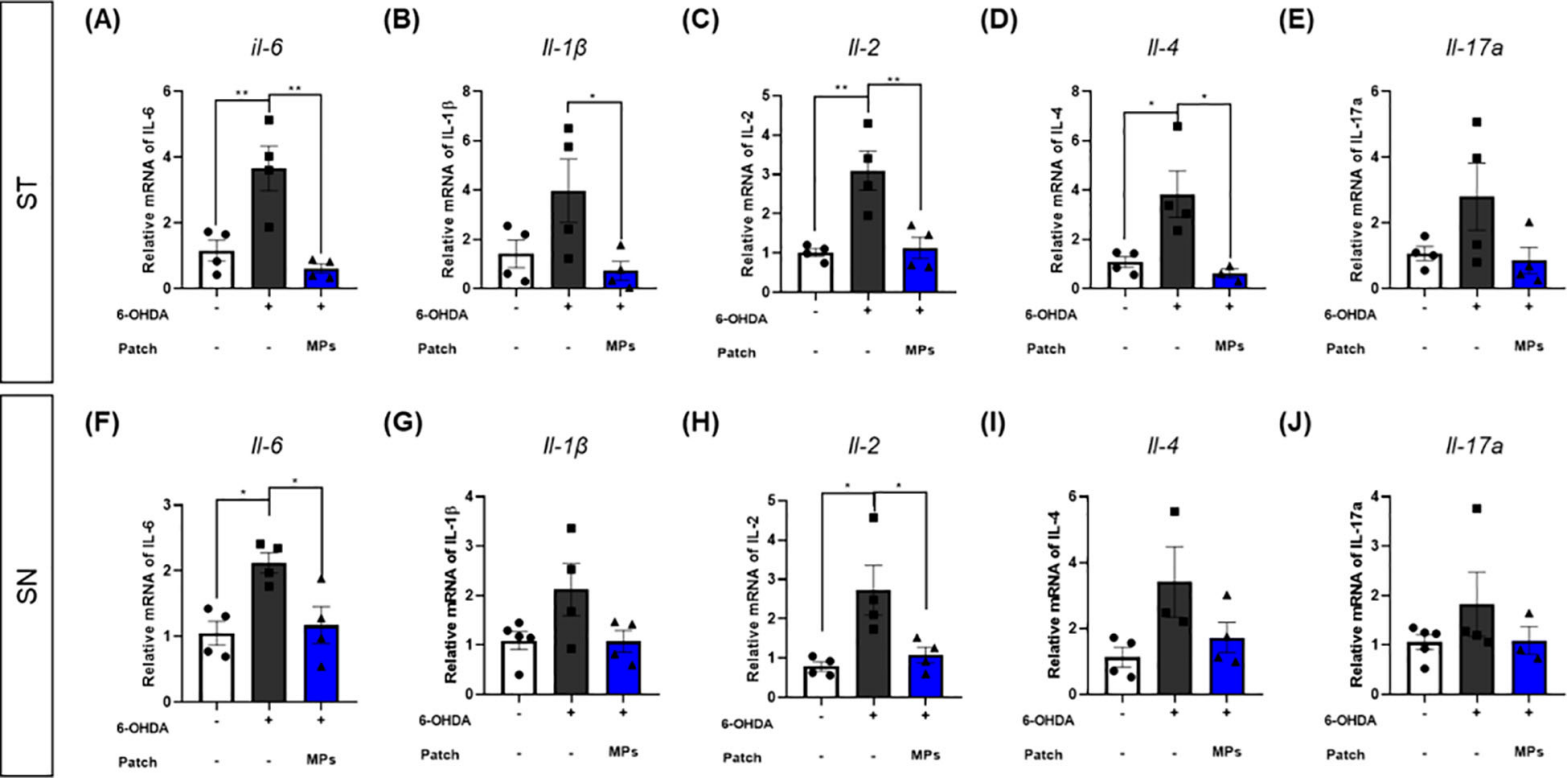
Effects of MPs attached to acupoints on mRNA expressions of cytokines in the brain of 6-OHDA-injected mice. The mRNA expressions of IL-6, IL-1β, IL-2, IL-4 and IL-17a in ST **(A–E)** and SN **(F–J)** were measured by qRT-PCR. Values are given as the mean ± S.E.M. Data were analyzed by One-way ANOVA followed by *post hoc* Dunnett’s multiple comparisons test. *p < 0.05 and **p < 0.01 compared to the 6-OHDA group using One-way ANOVA.

Cytokines contributing to the differentiation and activation of immune cells are also associated with neuronal death (37). Therefore, we assessed the mRNA expression of cytokines associated with B and T cells in the ST and SN. In both ST and SN, the mRNA expression levels of IL-6 (ST, **p < 0.01; SN, *p < 0.05), IL-1β (ST, p = 0.1; SN, p = 0.08), IL-2 (ST, **p < 0.01; SN, *p < 0.05), IL-4 (ST, *p < 0.05; SN, p = 0.05), and IL-17a (ST, p = 0.16; SN, p = 0.32)were increased by 6-OHDA injection. In contrast, in the MPs group, the levels of all cytokines were lower than those in the 6-OHDA group. In particular, the decreases in IL-6 (ST, **p < 0.01; SN, *p < 0.05), IL-1β (ST, *p < 0.05; SN, p = 0.09), IL-2 (ST, **p < 0.01; SN, *p < 0.05) and IL-4 (ST, *p < 0.05; SN, p = 0.09) in the ST and IL-6 and IL-2 in the SN were significant (IL-17a: ST, p = 0.11; SN, p = 0.42; **Figure 6**).

### 3.6 MP attachment to acupoints balancing peripheral immune system in the blood of 6-OHDA-induced PD mice

We further measured the number of immune cells in the blood to investigate whether the same effect was observed in the peripheral immune system connected to the skin where the MPs were attached. Changes in the T and B cells in the periphery of patients with PD have been reported in many cases (11, 38). The previously mentioned CD3+, CD4+, and CD8+ T cells enter the brain from the periphery when the BBB collapses. In addition, B cells activated in the periphery due to α-synuclein can induce the secretion of antibodies that are deposited in the center nervous system (39). Therefore, the normalization of peripheral immune cells in PD may have a direct effect on neuroinflammation within the brain.

**Figure 7:**
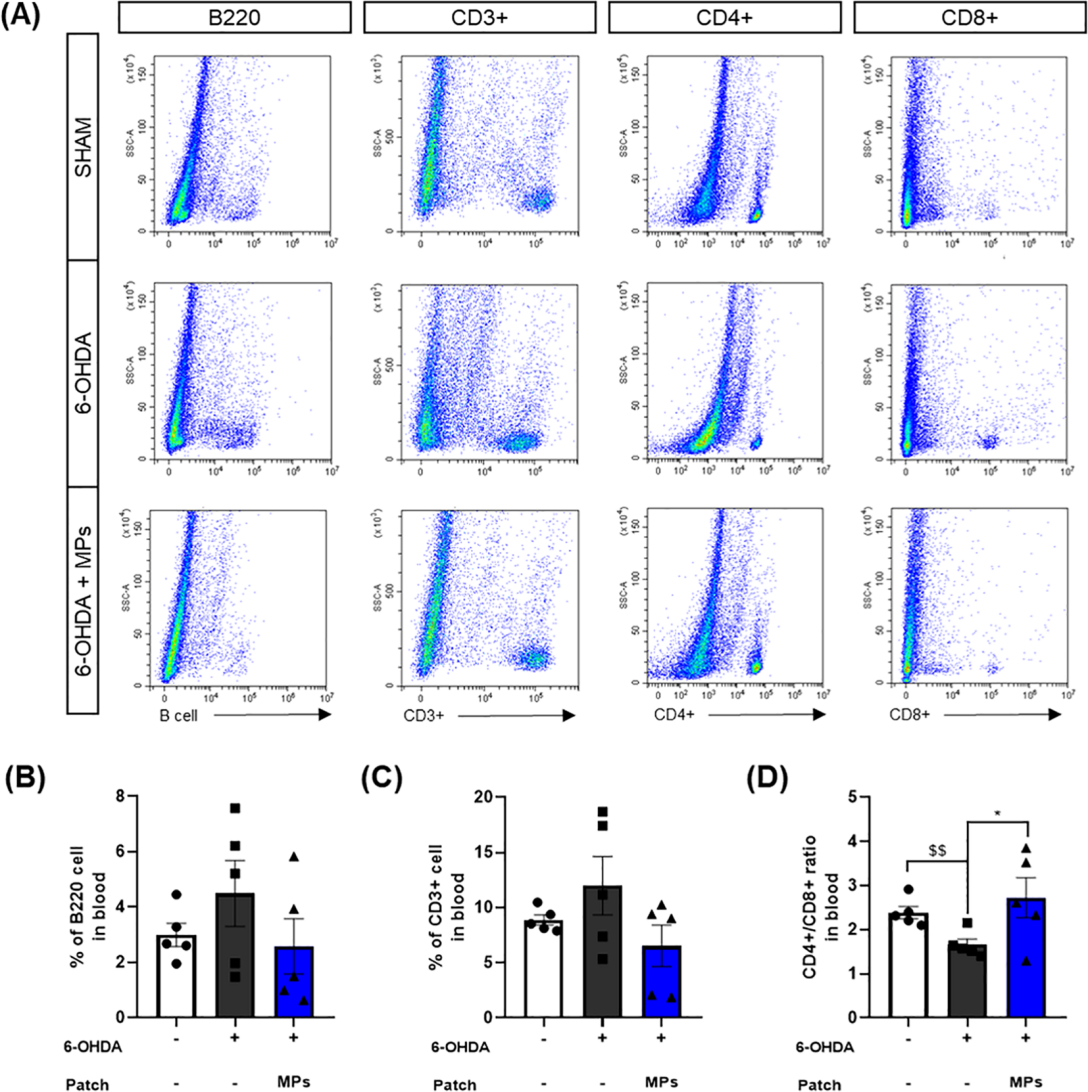
Effects of MPs attached to acupoints on % of B220, CD3+, CD4+ and CD8+ cells in the blood of 6-OHDA-injected mice. Representative flow cytometry plots of B220, CD3+, CD4+ and CD8+ cells in blood are shown in **(A)**. Graphs were represented as % of B220 **(B)**, CD3+ **(C)** and CD4+/CD8+ ratio **(D)** in blood. Values are given as the mean ± S.E.M. Data were analyzed by One-way ANOVA followed by *post hoc* Dunnett’s multiple comparisons test or Student’s *t*-test. *p < 0.05 compared to the 6-OHDA group using One-way ANOVA; $$*p* < 0.01 using student’s *t*-test.

As in the brain, the percentages of B220 B cells and CD3+ T cells, and the CD4+/CD8+ ratio were assessed in the blood. Injection of 6-OHDA was found to increase the percentages of B220 (p = 0.44) and CD3+ (p = 0.43) cells, and a decreased CD4+/CD8+ ratio in the blood ($$p < 0.01 in Student’s *t*-test), similar to the results in the brain. This suggests that the intrastriatal injection of 6-OHDA disrupts the peripheral immune system. In the MPs group, the percentages of B220 (p = 0.29) and CD3+ (p = 0.11) cells were lower and the ratio of CD4+/CD8+ cells was higher (*p < 0.05) in the blood than in the 6-OHDA group (**Figure 7**). These results suggest that MPs attached to acupoints restore the central and peripheral immune systems.

## 4 Discussion

In this study, we demonstrated that the attachment of MPs to GB20 and GB34 restored the immune system from the periphery to the brain, further inhibiting neuroinflammation and dopaminergic damage in the brain. Additionally, these tissue changes improved the behavioral disorders caused by 6-OHDA injection. We found that the attachment of MPs normalized the percentages of B220 and CD3+ cells and the ratio of CD4+ helper T cells/CD8+ killer T cells in the blood and brain, thus regulating cytokine levels in the brain. Furthermore, MPs attached to acupoints have been shown to inhibit the activation of microglia and astrocytes in the brain and regulate Bcl2/Bax and Nrf2/HO-1. These results indicate that the attachment of MPs to GB20 and GB34 could be a new treatment method for effectively improving PD pathology by mediating the immune system.

Acupoints have been identified in humans, and numerous studies have explored their corresponding locations in animals to facilitate effective research. Various studies have demonstrated that many acupoints in humans correspond to locations in mice (40, 41). Among these points, GB20 is located near the skull, close to the cervical nerves that connect the brainstem and upper spinal cord, and is known to regulate upper nervous system functions (42). Conversely, GB34 is situated at the junction of muscles and tendons and is involved in controlling lower body movement (42). Previous studies have also shown that simultaneous stimulation of both GB20 and GB34 is more effective than stimulating either point alone (43, 44). Based on this evidence, we selected GB20 and GB34 for microneedle stimulation in our study.

According to various studies, immune system dysfunction is prominent in PD, and is closely associated with neuroinflammation, a major pathology of PD (9, 45). It has been reported that aging, the primary risk factor for developing PD, leads to an increase in immune cells, but a decrease in their function (5). Among the variety of related immune cells, the most notable immune change in PD is a decrease in the CD4/CD8 ratio within T cells. In our study, 6-OHDA injection resulted in a decrease in the CD4+/CD8+ ratio in the brain and blood, as well as an increase in T and B cells. Additionally, we observed an increase in cytokine levels and the activation of microglia and astrocytes in the brain. This finding suggests that the 6-OHDA injection model is suitable for evaluating the effects of inflammation caused by immune disorders in patients with PD.

Additionally, our study demonstrated that the attachment of MPs to GB20 and GB34 normalized the levels of cytokines, B220, and CD3+, and the CD4+/CD8+ ratio caused by 6-OHDA injection in the blood and brain. The MPs attached to the skin at GB20 and GB34 are expected to cause changes in the peripheral immune system, which can further affect the immune system in the brain. Under normal circumstances, the peripheral and central immune systems are separate; however, in PD, the breakdown of the BBB leads to a mingling of the central and peripheral immune systems (39). Therefore, the regulation of peripheral immune cells can affect the brain more easily. This suggests that under normal conditions, the attachment of MPs does not cause significant changes to the brain immune system, and only has a significant effect under pathological conditions. In addition, MP-induced immune normalization reduces cytokine levels and suppresses the activation of microglia and astrocytes in the brain. Therefore, our results suggested that MPs suppress neuroinflammation in the brain via immune regulation.

Further, the current study showed that attachment of MPs to GB20 and GB34 prevented 6-OHDA-induced death of dopaminergic neurons specifically in the SN rather than the ST. In our study, 6-OHDA was injected into the ST, where terminal regions of dopaminergic neurons vulnerable to damage were clustered (46). As such, damage to the ST is more fatal, and recovery takes longer period of time. In our findings, there was no change in TH-optical density in the ST of the MPs group compared to that of the 6-OHDA group, but dopamine levels and DAT-optical density tended to increase in the MPs group. These results indicate that MPs may not have an effect on dopaminergic neurons in the ST, but that they might recover if MPs are applied for a longer period of time.

We found that the PD symptom improvement, dopaminergic neuron protection, and neuroinflammation inhibition effects observed in the MPs group did not occur in the SPs group. This result disproves that the PD improvement effect occurs due to microneedle acupoint stimulation rather than other effects. Our results suggest that MPs can effectively stimulate acupoints, which are key locations for traditional treatments. Therefore, this study is a new attempt to combine traditional and modern technologies.

This study had several limitations. First, it is unclear how MPs attached to the skin alter the peripheral immune system. Previous studies have further shown that acupuncture stimulation of acupoints activates mast cells, resulting in an immune cell response (47, 48). We expect that the immune response to acupuncture occurs similarly to the attachment of MPs, and we plan to analyze the mechanism through further study. Second, this study only selected GB20 and GB34 among many acupoints, and did not compare them with other acupoints. In addition to the GB20 and GB34 acupoints that we selected, other acupoints have been shown to be effective in treating PD, and comparative studies are needed.

Collectively, our results reveal that the attachment of MPs to GB20 and GB34 regulates the immune system from the periphery to the brain, suppressing neuroinflammation and protecting dopaminergic neurons in the brain. Therefore, the stimulation of acupoints using MPs has great potential as a candidate treatment for various diseases, including PD.

## Data Availability

The raw data supporting the conclusions of this article will be made available by the authors, without undue reservation.
